# Is the collateral circulation pattern in the hard palate affected by cleft deformity?

**DOI:** 10.1007/s00784-024-05627-0

**Published:** 2024-04-26

**Authors:** Arvin Shahbazi, Andreas A. Mueller, Szilvia Mezey, Sebastian Gschwindt, Tamás Kiss, Gábor Baksa, Reha S. Kisnisci

**Affiliations:** 1 Department of Anatomy, Histology and Embryology (Oral Morphology Group), Semmelweis University, Budapest, Hungary; 2 Department of Restorative Dentistry and Endodontics, Semmelweis University, Budapest, Hungary; 3 Department of Periodontology, Semmelweis University, Budapest, Hungary; 4 Department of Oral and Craniomaxillofacial Surgery, University Hospital Basel and University Children’s Hospital Basel, Basel, Switzerland; 5 Facial and Cranial Anomalies Research Group, Department of Biomedical Engineering and Department of Clinical Research, University of Basel, Basel, Switzerland; 6 Department of Biomedicine, University of Basel, Basel, Switzerland; 7 Pont32 Dental and Oral Surgery Clinic, Budapest, Hungary; 8 Department of Oral and Maxillofacial Surgery, Lokman Hekim University, Ankara, Türkiye; 9 Cleft Lip & Palate and Related Anomalies Research and Treatment Center, Ankara, Türkiye

**Keywords:** cleft palate, anastomoses, flaps, microcirculation, wound healing

## Abstract

**Objectives:**

To evaluate the influence of collateral vascularization on surgical cleft palate closure and deformities.

**Materials and methods:**

Corrosion casting was performed using red-colored acrylic resin in twelve fresh adult cadavers with a normal hard palate. Additionally, white-colored barium sulfate was injected into a fetus with a unilateral complete cleft palate, and layer-by-layer tissue dissection was performed. Both substances were injected into the external carotid arteries. Corrosion casting involved dissolving the soft and hard tissues of the orofacial area utilizing an enzymatic solution.

**Results:**

In normal palates, bilateral intraosseous infraorbital arteries formed a network in the premaxilla with the intraosseous nasopalatine- and greater palatine arteries (GPAs). The perforating GPAs anastomosed with the sphenopalatine artery sub-branches. Bilateral extraosseous GPA anastomoses penetrated the median palatine suture. Complex vascularization in the retrotuberal area was detected. In the cleft zone, anastomoses were omitted, whereas in the non-cleft zone, enlarged GPAs were distributed along the cleft edges and followed the anatomical course anteriorly to initiate the network with facial artery sub-branches.

**Conclusions:**

The anatomical subunits of the palate exhibited distinct anastomosis patterns. Despite omitted anastomoses with collateral circulation in the cleft zone, arteries maintained their anatomical pattern as seen in the normal specimen in the non-cleft zone.

**Clinical relevance:**

Based on the findings in normal- and cleft palates, surgeons may expect developed anastomosis patterns in the non-cleft zone. Due to the lack of microcirculation in the cleft zone, the existent anastomoses should be maintained as much as possible by the surgical technique. This applies anteriorly in the incisive canal territory, alveolar ridges, and posteriorly in the retrotuberal area.

## Introduction

Blood circulation plays a fundamental role in surgical healing, tissue growth, and remodeling. Therefore, mucoperiosteal and extra-, as well as intraosseous anastomoses perform a critical function in cleft palate surgeries. Common adverse events in cleft surgery, such as infection, fistula formation, tissue necrosis, scarring, and growth restrictions, might be triggered by impaired circulation. The blood supply of distinct parts of the palate follows the different embryological origins and developmental patterns of the hard and soft palate [1]. During development, the maxilla is primarily supplied by the stapedial artery, which undergoes involution, and the vascularization shifts to the parapharyngeal branches from the external carotid artery (ECA) [2]. Later, the maxillary artery (MA) branches mainly supply the mature hard palate. Multiple developmental contributions to the soft palate result in abundant vascularization and anastomoses, with the participation of vessels proximal to the MA [3, 4]. Thus, branches of the ascending palatine artery (APA) and the ascending pharyngeal artery (APHA) are crucial in supplying the maxillary-alveolar segment in the case of Le-Fort I osteotomies [5].

Prenatal developmental abnormalities may affect local vascularization and influence tissue organization along the cleft margin [3, 4]. Depending on the type of cleft formation, several anastomoses fail to develop, which reduces the alternatives of collateral blood flow postoperatively. Further to that, the surgical incisions, as well as potential hematoma and inflammation, reduce microcirculation. Additional negative effects on circulation with a risk of tissue damage might arise from tissue tension by sutures or palatal dressing plates, or tissue compression by extensive transposition. These complications are more prone if the cleft is wide and the quantity of soft tissue is underdeveloped. Therefore, understanding the vascular pattern in the cleft surgical territory helps to preserve the microcirculation, which is a key factor for healing.

Previously, Bosma [6] described the abundant vascular network in the palate anteriorly-laterally across the alveolar ridge with the vestibular branches of the facial artery (FA) in infants. Recently, Shahbazi et al. [7] demonstrated various anastomosis patterns among branches of the greater palatine artery (GPA) with the lesser palatine arteries (LPA) and nasopalatine artery (NPA) in the hard palate. These anastomoses can convert into a functional circulation when the GPA is injured during palatoplasty to ensure healing and prevent tissue necrosis. Likewise, the typical course of the vessels within the tissue layers must be understood to define optimal soft tissue handling and selection of dissection planes during cleft repair. This specifically applies to the LPA branches that supply the minor salivary glands, predominantly at the posterior aspect of the hard palate [8, 9]. Preserving circulation through these arterial anastomoses is essential in cleft palate surgery to ensure safe healing and minimize scarring. An accurate understanding of the developmental, topographical, and functional aspects of the vascular territories is required to devise surgical strategies for cleft repair with minimal repercussions on vascular-related risk for healing and growth. Therefore, the aim of this study was to investigate mucoperiosteal, as well as extraosseous and intraosseous anastomoses in the territory of hard palate cleft surgery, in order to delineate the relevant collateral circulation for various incision outlines.

## Materials and methods

Twelve (7 males, 5 females; 55-90 years of age) adult human cadavers without arterial comorbidities were selected for the corrosion casting study. A spontaneously aborted fetus (1 male, 26 weeks of intrauterine age) with an incomplete alveolar cleft and a unilateral complete hard and soft palate cleft was investigated to detect the arterial pattern by injection of barium sulfate. The cadavers were donated for scientific purposes to the Department of Anatomy, Histology and Embryology, Semmelweis University, Budapest, Hungary, according to Hungarian laws for anatomical donation (approval number: 110/2020.(VII.07.)). This investigation was conducted following the Declaration of Helsinki.

In the adult cadavers, the macroscopic arterial path in the hard palate and territory was stained via corrosion casting. The ECAs were isolated and irrigated with saline solution. The mixture of acrylic resin (ACRIFIX 190 (2 R 0190), Evonik Industries AG., Germany) and red Akemi Akepox coloring agent (AKEMI GmbH., Nürnberg, Germany) was injected into the ECA. For complete polymerization of the mixture in the arteries, the specimens were retained for 24 hours at room temperature. Afterward, the donor heads were kept in an enzymatic solution (Somat gold 12 actions (Henkel AG., Germany)), which was replaced every 15-20 days at 36°C for about 60 days to macerate soft tissues. In the next step, the detritus and remnants were washed out using tap water. Then, the samples were stored in cold water for about three days to clear the residual chemicals. After visualizing the anastomoses of the extraosseous branches related to the hard palate, the intraosseous branches were detected by precise local bone dissolution with 2-4% potassium hydroxide (KOH).

In the fetus specimen, the ECAs were canulated and washed with saline solution after dissection and isolation. The white-colored barium sulfate agent (BaSO4) (Micropacque®, Guerbet GmbH, Sulzbach/Taunus, Germany) was injected into ECAs. The specimen was fixed in a paraformaldehyde solution. The vascular route around the cleft area was dissected.

## Results

**Fig. 1:**
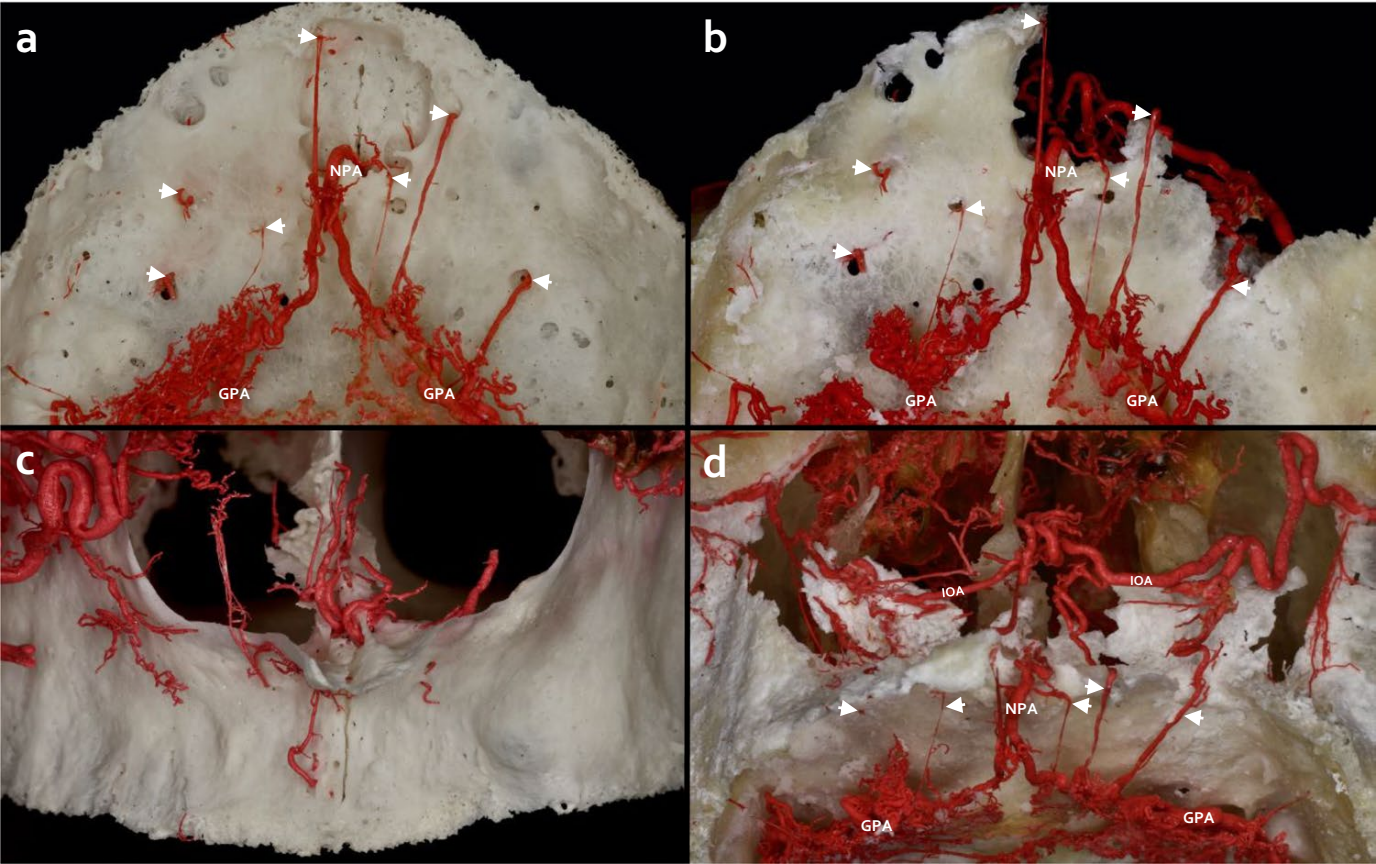
Intraosseous arterial anastomoses of the premaxilla. **a**) Premaxilla (inferior view) without maceration of the hard tissue, arrows (→) indicate the intraosseous branches of the greater palatine arteries (GPAs) and the nasopalatine artery (NPA). **b**) After hard tissue maceration, the premaxilla (inferior view) displays the intraosseous branches of the GPA/NPA that establish anastomoses with the infraorbital artery (IOA). **c**) Premaxilla (anterior view) without hard tissue maceration, the arterial network emerging around the anterior nasal spine. **d**) Premaxilla (anteroinferior view) with hard tissue maceration, the bilateral IOAs form complex anastomoses with the GPA/NPA

**Fig. 2:**
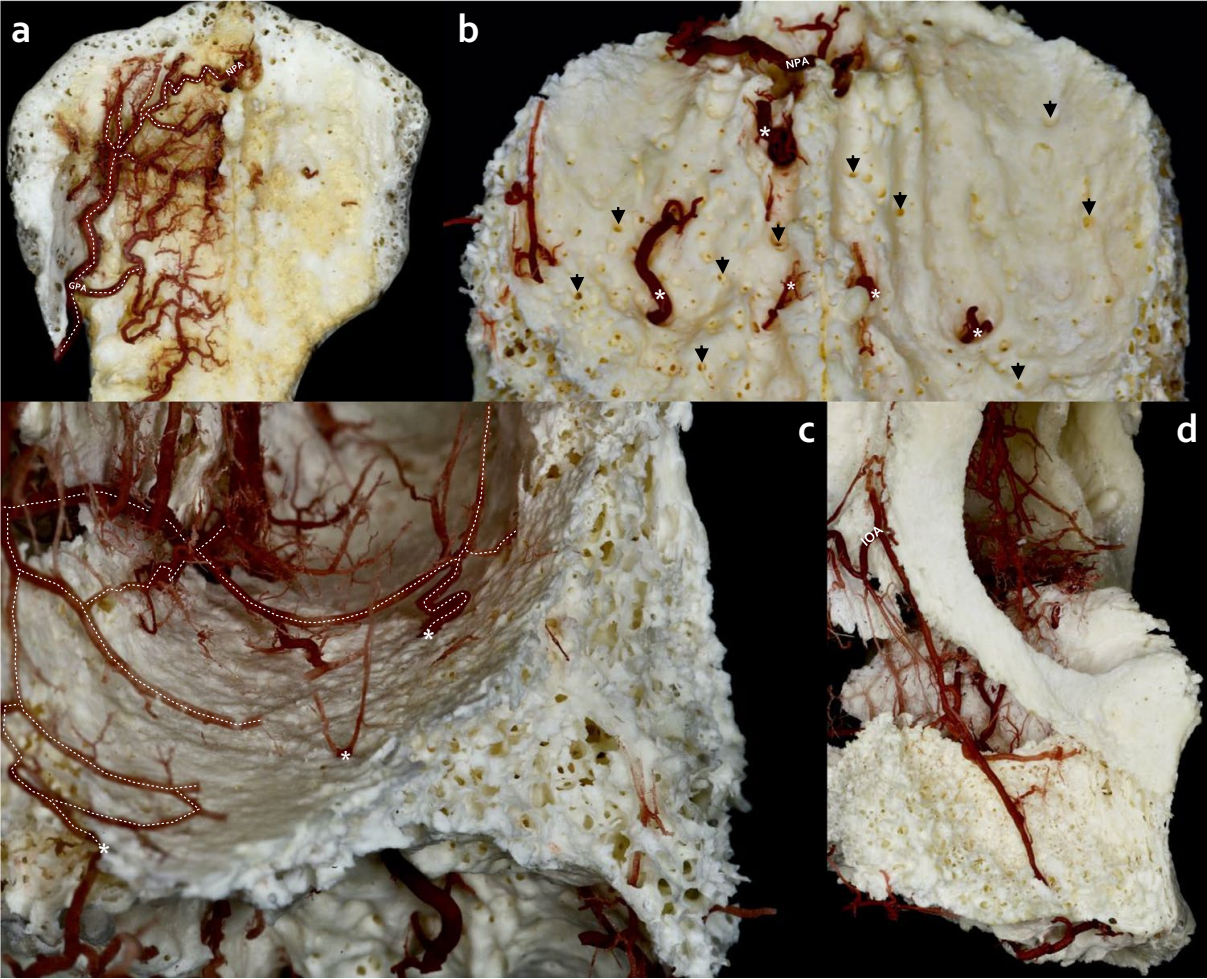
Anastomoses pattern of the hard palate with the nasal cavity and maxilla. **a**) Overview of extraosseous mapping of the greater palatine artery (GPA) branches with anastomosis to the nasopalatine artery (NPA). **b**) After removing extraosseous branches of GPA osseous branches (*) are noticed in the anterior and middle aspects of the hard palate with several bony openings labeled with arrows (↓). **c**) Posterior view from the floor of the nasal cavity, perforating branches (*) of GPA forming anastomoses with the branches of the posterior septal nasal branches of the sphenopalatine artery. **d**) Anterolateral view of right maxilla, intraosseous branch of the infraorbital artery (IOA) supplies mainly the alveolar ridge territory, establishing collateral circulation between the maxilla and hard palate

**Fig. 3:**
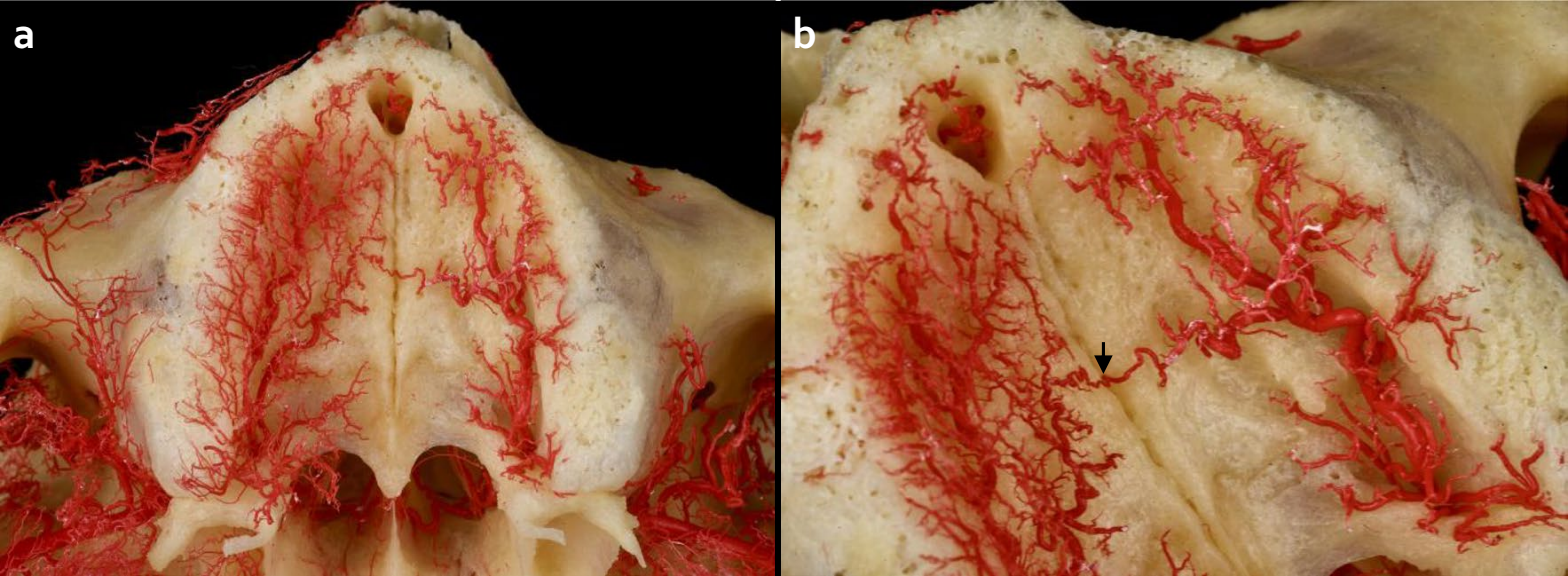
Determination of penetrating intraosseous branch at the midpoint of the hard plate in the median palatine suture. **a**) Overview of hard palate arterial supply with bilateral anastomosis between greater palatine arteries (GPAs). **b**) A direct penetrating branch (↓) from bilateral anastomosis of GPAs entering median palatine suture

**Fig. 4:**
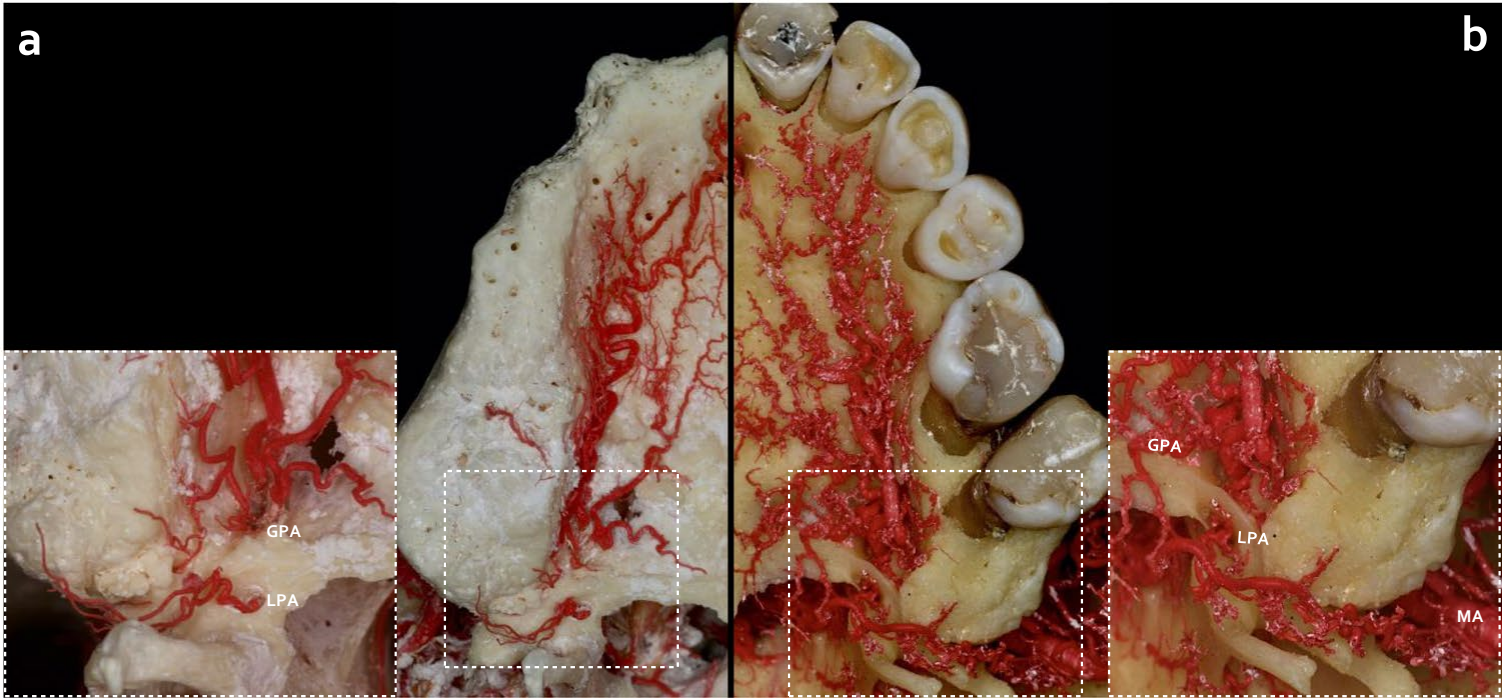
Retrotuberal arterial pattern. **a**) The retrotuberal aspect of maxillary tuberosity received an intraosseous branch from the greater palatine artery (GPA) and an extraosseous branch from the lesser palatine artery (LPA). **b**) The retrotuberal aspect of maxillary tuberosity presented complex extraosseous anastomoses between branches of GPA, LPA, and maxillary artery (MA), respectively

In all adult cadavers both intra- and extraosseous anastomosis patterns were detected in the hard palate. From the anterior to the posterior direction, these are (Figs. 1, 2, 3, 4):

- Intraosseous anastomoses between bilateral infraorbital arteries (IOAs) were detected at the level of the intermaxillary suture (Fig. 1). The network between IOA-NPA/GPA sub-branches perfused the alveolar ridge in the region of the premaxilla (Figs. 1 and 2).
- Perforating GPA branches on the anterior/middle aspect of the palatine process of the maxilla anastomosed with the posterior septal nasal branches of the sphenopalatine artery (SPA) at the floor of the nasal cavity (Fig. 2).
- Penetrating arteries as part of an anastomosis between branches of bilateral GPAs infiltrated the median palatine suture (Fig. 3).
- Extraosseous retrotuberal anastomoses among the GPA, LPA, and MA branches were formed between the maxillary tuberosity and the pterygoid hamulus (Fig. 4).

**Fig. 5:**
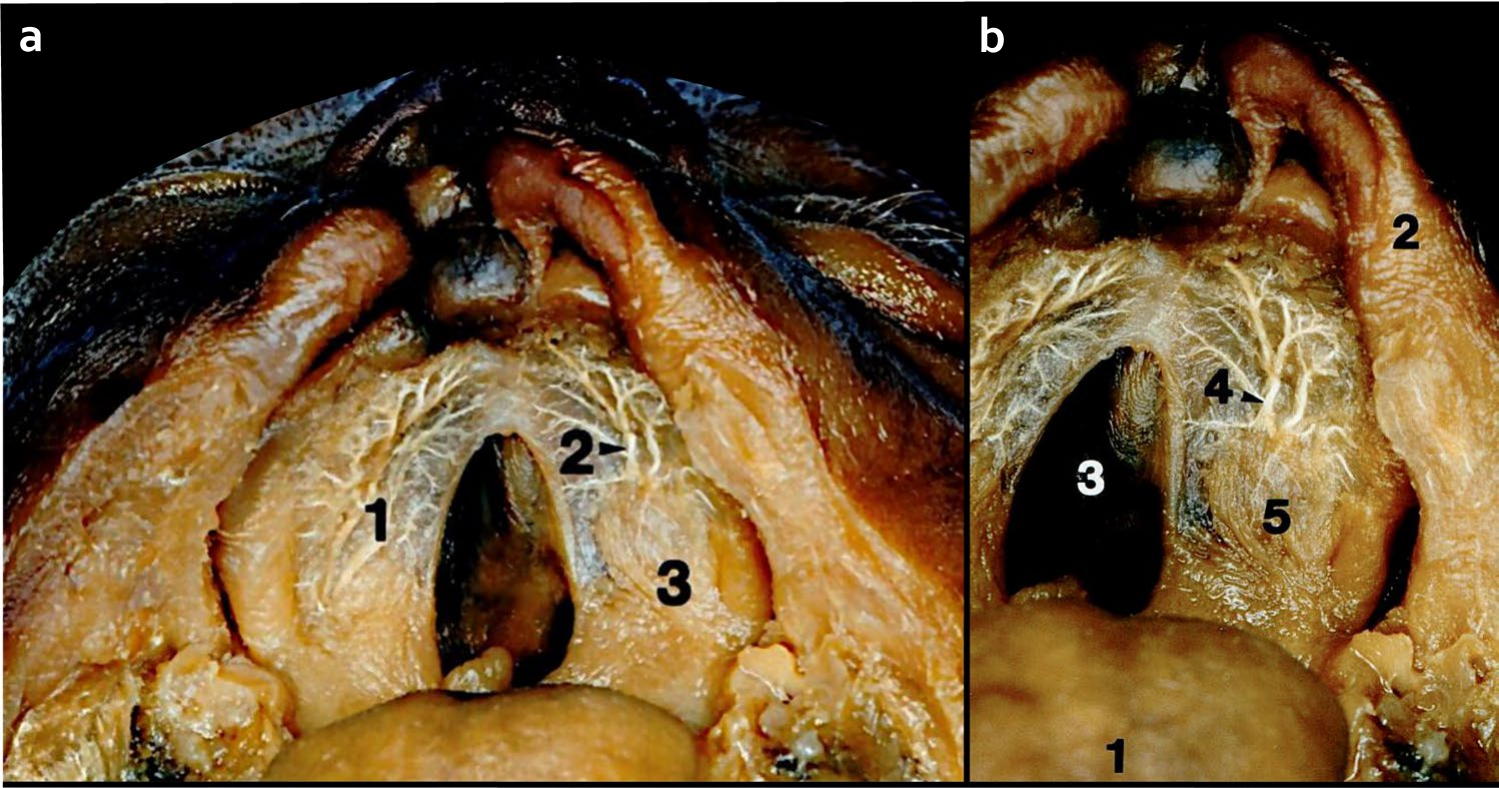
Fetus with an incomplete alveolar cleft with unilateral complete hard and soft palate cleft, injected with barium sulfate. **a**) Overview of the cleft palate, arterial distribution of the greater palatine artery (GPA) after mucosal dissection where contralateral anastomoses are omitted. 1- Hard palate; 2- GPA; 3- Fibers of soft palate muscle **b**) The allocation of GPA sub-branches in developed and unaffected areas, the vascularization in non-affected zones is maintained, particularly in the anterior palato-premaxillary territory. 1-Tongue; 2- Upper lip; 3- Cleft zone; 4- Non-cleft zone with GPA sub-branches; 5- Fibers of soft palate muscle

In the fetal specimen, the vascularization pattern of the cleft and the non-cleft zones were analyzed (Fig. 5):

- In the non-cleft zone, GPA sub-branches followed the anatomical path. Particularly in the anterior aspect of the hard palate, arteries shifted towards the vestibule to initiate extraosseous anastomoses with the FA sub-branches.
- In the non-cleft zone, the dimensions of arteries appeared to be enlarged and distributed to the edge of the cleft.
- In the cleft zone, the bilateral GPA anastomoses were predominantly neglected, and the perforating and penetrating branches were omitted.

## Discussion

Vascularization of the hard palate embryologically relies on the extension, elevation, and contact of the secondary palatal shelves, as well as the fusion of the primary and secondary palates. Developmental failures disrupt the blood supply and result in underdeveloped tissue due to disorganization of the vasculature at the margin of the cleft [4]. Several angiogenesis-related genes correspondingly influence the development of the palate, suggesting that the formation of its supplying vessels follows behind the formation of the hard palate [10]. Therefore, the presence of blood vessels is not thought to be related to the action of elevation and closure of the palate [11, 12].

Studies on cadavers have shown that extensive vascularization and anastomoses may permit the hard palate and alveolar ridges to receive collateral circulation [5–7, 13–16]. In the current investigation, bilateral intraosseous anastomoses between IOAs at the level of the anterior nasal spine/ intermaxillary suture were observed. Additionally, multiple intraosseous anastomoses between IOA-GPA/IOA-NPA were detected in the premaxilla, which might be deficient due to alveolar cleft or, in case of non-alveolar cleft, may have a crucial impact on the circulation during tissue mobilization in the vicinity of the incisive canal. The formation of these anastomoses above the anterior aspect of the hard palate, as described in this study, is likely to occur during the fusion of the two lateral palatal shelves. This phenomenon occurs as the medial edge epithelial cells of opposing shelves adhere to one another to form the midline epithelial seam and then migrate into deeper regions, undergoing epithelio-mesenchymal transformation and guiding the blood vessels with them [17]. According to previous [7, 13, 15] and present studies, the front/mid aspects of the hard palate and alveolar ridges contain increased intraosseous vascularization, which might be torn off during full elevation of the posterior pedicled flap [18–20]. Furthermore, the osseous perforating anastomoses in the premolar-canine region between GPA-posterior septal nasal branches of SPA and GPA-IOA, as well as a bony penetrating branch from the anastomosis between bilateral GPAs traversing the median palatine suture highlights the changed microcirculatory anastomosis pattern during cleft surgeries. Our observations in the fetal unilateral cleft palate arterial analysis, along with the results reported by Wilhelm [16], indicate that extra- and intraosseous anastomoses, as well as collateral blood flow in the cleft anatomy, can be altered depending on the extension of the deformity. Nevertheless, in the ordinarily developed areas, the vascular pattern follows the anatomical path.

**Fig. 6:**
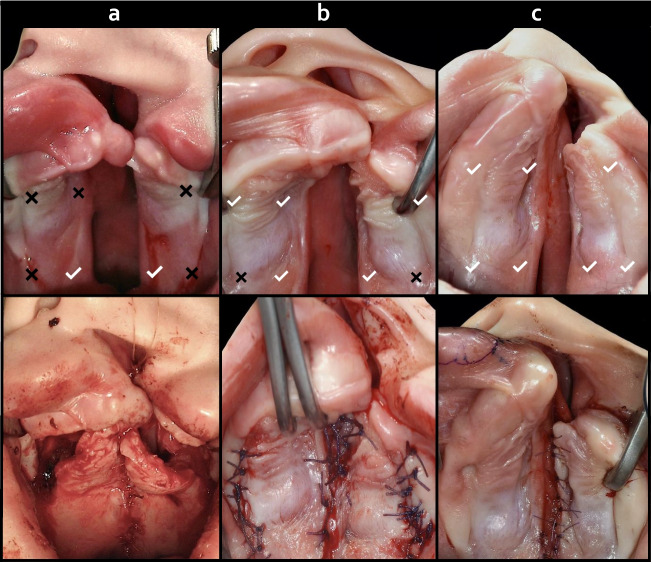
Comparative change of vascular collateral supply in various techniques of palate cleft surgery (preoperative (above) and postoperative (below) aspects of each patient). Collateral zones are labeled as affected (✘) or preserved (✔) at critical regions: Anterolateral GPA-IOA-FA, Anteromedial GPA-NPA, Posterolateral GPA-LPA-PSAA-MA, Posteromedial GPA-LPA-APA-APHA. **a**) Uni-pedicle flap. **b**) Bi-pedicle flap. **c**) Minimal incision palatoplasty (combined with continuous circular closure). (*Courtesy of AAM*). Greater palatine artery (GPA); Infraorbital artery (IOA); Facial artery (FA); Nasopalatine artery (NPA); Lesser palatine artery (LPA); Posterior superior alveolar artery (PSAA); Maxillary artery (MA); Ascending palatine artery (APA); Ascending pharyngeal artery (APHA)

The palatal cleft can be closed in distinct fashions depending on its localization and structure, which can be complete or incomplete and unilateral or bilateral, respectively. Von Langenbeck [21] was the first to devise a reproducible hard palate repair technique by utilizing subperiosteal dissection and bilateral releasing incisions lateral to the GPA from the tuberosity towards the anterior alveolar ridge. Midline suture was performed on the oral layer only creating bi-pedicled mucoperiosteal flaps. In contrast, the mucoperiosteum was preserved intact behind the anterior alveolar ridge, resulting in a residual opening. Since then, cleft surgical techniques have evolved in multiple aspects. However, a generally accepted classification system does not exist today. Concerning the anatomical vascular landmarks in the hard palate, techniques being frequently used today can be assigned to the following major categories (Fig. 6): i) Uni-pedicle (posterior based) vs. bi-pedicle (anterior/posterior based) palatal mucosa supply ii) Uni-layer (oral or nasal) vs. bi-layer (oral and nasal) mucosal closure in the midline iii) Medial incision (minimal) vs. combined incisions (medial and lateral) for surgical access iv) Straight line vs. transverse incisions techniques at the hard-soft palate junction.﻿

In standard anatomical circumstances, an incision designed unilaterally on the palate may receive supply from the contralateral side to heal [7]. Conversely, in palatal clefts the contralateral supply is absent and the surgical incision must rely on the ipsilateral collaterals to heal. Therefore, incisions should be limited and anterior collateral vascularization should be maintained [22]. In addition, in bilateral clefts, the circulation in the anterior palate is further diminished by the absence of the NPA-GPA anastomosis; thus, the distinct vascular territories of the respective cleft malformation must be kept in mind for incision and dissection during primary and secondary cleft surgeries. Reports on cleft surgical techniques repeatedly warranted from maneuvers to increase flap mobility by lateral releasing incisions toward the posterior aspect of the maxillary tuberosity at the junction between the supraperiosteal - subperiosteal planes and splitting off the hamulus [23–26]. Disturbed blood circulation and detrimental scarring in this area from secondary healing is accused to impair facial growth.

Gauthier et al. [27] investigated anatomically the mucosal perfusion of the palate by ligating the descending palatine artery (DPA). They discovered that although the supply of mucosa was diminished, the tissue vascularization was maintained due to the collateral branches of the APA and APHA. Nevertheless, the alternate vascularization can show individual variations with a risk of insufficient co-supply from case to case [5, 27]. The variations may account for the controversial findings about the DPA for perfusion of the maxilla. A clinical randomized human trial found no statistical difference in gingival microcirculation at the apex of the front tooth in Le Fort I osteotomy related to DPA ligation [28]. However, osseo-mucosal avascular necrosis can be a distressing complication based on the reports in patients with a history of cleft palate repair followed by additional fistula repair [29, 30].

The current investigation has also shown the existence of a critical arterial network between the maxillary tuberosity and the pterygoid hamulus territories, formed by the branches of the GPA, LPA, and MA, which confirms the avoidance of retrotuberal incisions/flaps, especially in primary cleft surgery.

## Conclusion

The hard palate displays various configurations of arterial anastomoses. Cleft palate deformity predominantly reduces vascular anastomoses and collateral circulation in the cleft zone; however, vascularization in the normally developed palatal areas maintain the anatomical pathway. Therefore, the dissection strategies should maximally respect the remaining anastomoses in order to preserve adequate microcirculation for proper healing and tissue development.

## References

[1] Dixon MJ, Marazita ML, Beaty TH, Murray JC (2011). Cleft lip and palate: Understanding genetic and environmental influences. Nat Rev Genet.

[2] Hinrichsen KV, Beier HM, Breucker H (2014). Humanembryologie: Lehrbuch und Atlas der vorgeburtlichen Entwicklung des Menschen. Aufl.

[3] Cohen SR, Chen L, Trotman CA, Burdi AR (1993). Soft-palate myogenesis—a developmental field paradigm. Cleft Palate Craniofac J.

[4] Cohen SR, Chen LL, Burdi AR, Trotman CA (1994). Patterns of abnormal myogenesis in human cleft palates. Cleft Palate Craniofac J.

[5] Bruneder S, Wallner J, Weiglein A, Kmečová Ĺ, Egger J, Pilsl U, Zemann W (2018). Anatomy of the Le Fort I segment: Are arterial variations a potential risk factor for avascular bone necrosis in Le Fort I osteotomies?. J Craniomaxillofac Surg.

[6] Bosma JF (1986). Anatomy of the infant head.

[7] Shahbazi A, Grimm A, Feigl G, Gerber G, Székely AD, Molnár B, Windisch P (2019). Analysis of blood supply in the hard palate and maxillary tuberosity-clinical implications for flap design and soft tissue graft harvesting (a human cadaver study). Clin Oral Investig.

[8] Mu L, Chen J, Li J, Fowkes M, Benson B, Nyirenda T, Sobotka S, Christopherson M, Sanders I (2021). Innervation of human soft palate muscles. Anat Rec (Hoboken).

[9] Liu J, Wang Y, Li H, Wu D, Song T, Yin N (2022). Vascular anatomy of the velopharyngeal muscles and its clinical implications: A fresh cadaveric study based on micro-computed tomography. Clin Anat.

[10] François-Fiquet C, Poli-Merol ML, Nguyen P, Landais E, Gaillard D, Doco-Fenzy M (2014). Role of angiogenesis-related genes in cleft lip/palate: Review of the literature. Int J Pediatr Otorhinolaryngol.

[11] Brinkley L, Basehoar G, Branch A, Avery J (1975). A new in vitro system for studying secondary palatal development. J Embryol Exp Morph.

[12] Brinkley L, Basehoar G, Avery J (1978). Effects of craniofacial structures on mouse palatal closure in vitro. J Dent Res.

[13] Shahbazi A, Feigl G, Sculean A, Grimm A, Palkovics D, Molnár B, Windisch P (2021). Vascular survey of the maxillary vestibule and gingiva-clinical impact on incision and flap design in periodontal and implant surgeries. Clin Oral Investig.

[14] Shahbazi A, Pilsl U, Molnár B, Feigl G (2020). Detection of Vascular Pathways of Oral Mucosa Influencing Soft- and Hard Tissue Surgeries by Latex Milk Injection. J Vis Exp.

[15] Shahbazi A, Sculean A, Baksa G, Gschwindt S, Molnár B, Vág J, Bogdán S (2023). Intraosseous arterial alteration of maxilla influencing implant-related surgeries. Clin Oral Investig.

[16] Wilhelm R (1969) Die chirurgische Anatomie der Gefäss- und Nervenversorgung des harten und weichen Gaumens bei Neugeborenen unter der Berücksichtigung operativer Eingriffe. Wissenschaftliche Zeitschrift der Friedrich-Schiller-Universität Jena/Thüringen:815–818

[17] Ferguson MW (1987). Palate development: mechanisms and malformations. Ir J Med Sci.

[18] Wardill WE (1928). Cleft Palate. Brit J Surg.

[19] Veau V, Borel S (1931). Division Palatine: Anatomie.

[20] Bardach J (1995). Two-flap palatoplasty: Bardach's technique. Oper Tech Plast Reconstr Surg.

[21] Von Langenbeck B (1861). Operation der angeborenen totalen Spaltung des harten Gaumens nach einer neuen Methode. Dtsch Klin.

[22] Benitez BK, Brudnicki A, Surowiec Z, Singh RK, Nalabothu P, Schumann D, Mueller AA (2022). Continuous circular closure in unilateral cleft lip and plate repair in one surgery. J Craniomaxillofac Surg.

[23] Kane AA, Lo LJ, Yen BD, Chen YR, Noordhoff MS (2000). The effect of hamulus fracture on the outcome of palatoplasty: a preliminary report of a prospective, alternating study. Cleft Palate Craniofac J.

[24] Mendonca DA, Patel KB, Skolnick GB, Woo AS (2014). Anatomical study of the effects of five surgical maneuvers on palate movement. J Plast Reconstr Aesthet Surg.

[25] Ogata H, Sakamoto Y, Kishi K (2017). Cleft Palate Repair without lateral relaxing incision. Plast Reconstr Surg Glob Open.

[26] Mommaerts MY, Gundlach KK, Tache A (2019). "Flip-over flap" in two-stage cleft palate repair. J Craniomaxillofac Surg.

[27] Gauthier A, Lézy JP, Vacher C (2002). Vascularization of the palate in maxillary osteotomies: Anatomical study. Surg Radiol Anat.

[28] Dodson TB, Bays RA, Neuenschwander MC (1997). Maxillary perfusion during Le Fort I osteotomy after ligation of the descending palatine artery. J Oral Maxillofac Surg.

[29] Teemul TA, Perfettini J, Morris DO, Russell JL (2017). Post-operative avascular necrosis of the maxilla: a rare complication following orthognathic surgery. J Surg Case Rep.

[30] Heggie A, Robertson K, Shand J (2021). Avascular necrosis in cleft maxillary repositioning: a review of cases and introduction of the 'delayed maxillary flap'. Int J Oral Maxillofac Surg.

